# Aortic Dissection With Pseudoaneurysm Formation Masquerading as Pulmonary Embolism After Transcatheter Aortic Valve Replacement

**DOI:** 10.1002/ccd.70148

**Published:** 2025-09-05

**Authors:** Heather Wang, Juan Bello, Dhir Gala, Jeffrey S. Lander

**Affiliations:** ^1^ Department of Medicine Rutgers New Jersey Medical School Newark New Jersey USA; ^2^ Cooperman Barnabas Medical Center Livingston New Jersey USA

**Keywords:** aortic aneurysm, Aortic dissection, pseudoaneurysm, pulmonary embolism, TAVR

## Abstract

Transcatheter aortic valve replacement (TAVR) is a commonly performed procedure for the treatment of severe aortic stenosis. While it is generally considered a low‐risk procedure, one of the rare potentially life‐threatening complications includes aortic dissection. We report the case of a 75‐year‐old immunocompromised female who presented 2 weeks post‐TAVR with persistent and worsening dyspnea, intermittent chest pain, and hypoxia. The patient's symptoms and risk factors increased the suspicion for pulmonary embolism, and initial imaging was supportive of the diagnosis. Management with therapeutic anticoagulation did not resolve the symptoms, and the patient developed acute‐onset anemia. Further investigation revealed an aneurysmal dilation and dissection of the ascending thoracic aorta with pseudoaneurysm causing near‐complete obstruction of the right pulmonary artery. This case highlights the diagnostic challenges of a rare and delayed post‐TAVR complication and describes a unique presentation of aortic dissection. Post‐operative complications should be considered in diagnosis even weeks after the procedure.

## 1 Introduction

Transcatheter aortic valve replacement (TAVR) is the most common method of replacing the aortic valve in patients with severe aortic stenosis [1]. Aortic dissection, a rare complication post‐TAVR, has an incidence rate of around 1.9%. It typically presents with new‐onset chest and back pain within 3 days post‐procedure [2]. While aortic dissection can rarely be associated with hematoma or aneurysm formation that can compress adjacent structures, such cases are infrequent and usually not associated with TAVR [3, 4]. We describe a rare case of a patient presenting 2 weeks post‐TAVR with dyspnea, hypoxia, and intermittent chest pain suggestive of pulmonary embolism, later diagnosed as aortic dissection with a pseudoaneurysm causing right pulmonary artery obstruction.

## 2 Case Report

A 75‐year‐old immunocompromised female presented to the emergency room with 2 weeks of worsening shortness of breath, intermittent chest pain, and progressive fatigue which rendered the patient mostly bedbound since the symptoms started. Previous medical history was significant for colon cancer treated with surgical resection, end‐stage renal disease due to post‐infectious glomerulonephritis requiring two kidney transplants, and an extensive cardiac history that included multi‐vessel coronary artery disease treated with coronary artery bypass grafting, atrial fibrillation (on apixaban 5 mg twice a day), and severe aortic stenosis.

Two weeks before presentation, the patient underwent an elective TAVR with a 23‐mm Edwards SAPIEN 3 valve (Edwards Lifesciences, CA, USA) for severe symptomatic aortic stenosis (peak gradient 65 mmHg, mean gradient 27 mmHg, aortic valve area 0.7 cm^2^). CT angiography revealed a calcium score of 1685 Agatston units and an aortic annular area of 374 mm^2^, with severe calcification and narrowing of the abdominal aorta. During the procedure, femoral access was obtained with placement of 6‐French and 8‐French sheaths in the right common femoral artery and vein. A 6‐French pigtail catheter was inserted into the aortic root to obtain a coplanar view. A 5‐French balloon‐tipped pacemaker was placed via the femoral vein into the right ventricular apex, and pacing was tested. An arterial cutdown was performed on the right subclavian artery, and a 6‐French sheath was inserted. After administering 5000 units of heparin intravenously, the aortic valve was crossed using an Amplatz Left (AL) 1 catheter and Terumo guidewire. The Terumo wire was exchanged for a Confida wire, and the AL1 catheter was replaced with a 14‐French Edwards eSheath. The 23‐mm SAPIEN 3 valve was deployed without complication, and proper valve placement was confirmed. Immediately post‐procedure, the patient developed sinus bradycardia with occasional sinus pauses and a left bundle branch block, which was managed with a temporary transvenous pacemaker placed via the right internal jugular vein, followed by a dual‐chamber pacemaker (Medtronic Azure XT DR W1DR01, Medtronic, NJ, USA) placed the following day. Post‐procedural transthoracic and transesophageal echocardiogram found trace paravalvular aortic regurgitation without a significant gradient across the valve. Throughout the hospitalization, the patient's blood pressure was stable with systolic blood pressures in the 120−130 s mmHg, with intermittent spikes into the 170 s mmHg which resolved with the addition of losartan 25 mg daily. Upon discharge, apixaban 5 mg twice a day was resumed and clopidogrel 75 mg daily started. The patient was discharged home several days later without complications.

On presentation 19 days later, the patient reported worsening dyspnea and intermittent chest pain. Initial vitals showed a blood pressure of 130/61 mmHg, heart rate of 66 beats per minute, respiratory rate of 22 breaths per minute, and an oxygen saturation of 73% on room air. Supplemental oxygen was started via high‐flow nasal cannula set at 15 L/min and 40% FiO2. The D‐dimer was elevated at 6531 ng/mL, high‐sensitivity troponin was 49 ng/L, and repeat troponin was stable (Table 1). An electrocardiogram showed ventricular pacing without ischemic ST‐segment changes. Chest X‐ray showed a small to moderate right pleural effusion. The patient's recent immobilization and age indicated a low‐to‐moderate pretest probability for pulmonary embolism using the revised Geneva Score and Wells’ Criteria. A lung ventilation‐perfusion scan was performed which demonstrated significantly reduced perfusion of the right lung relative to ventilation, findings concerning for a large pulmonary embolism. Given the previous history of multiple renal transplants, further imaging with CT angiography of the thorax was deferred.

**Table 1:** Initial laboratory results on admission.

Laboratory test	Value
*Chemistry*	
Sodium	140 mmol/L
Potassium	3.8 mmol/L
Chloride	107 mmol/L
CO2	26 mmol/L
Calcium	8.5 mg/dL
BUN	19.9 mg/dL
Creatinine	0.71 mg/dL
Total protein	4.3 g/dL
Albumin	2.9 g/dL
AST	20 Unit/L
ALT	9 Unit/L
Alkaline phosphatase	51 Unit/L
Bilirubin, total	1.0 mg/dL
*Cardiac profile*	
CK‐MB	1.7 ng/mL
Troponin T HS	49 ng/L
Troponin T HS 3 h	49 ng/L
BNP	247 pg/mL
*Hematology*	
WBC	13.8 K/CMM
RBC	3.83 MIL
Hgb	11.8 g/dL
Hct	35.6%
MCV	93.0 fL
MCH	30.8 pg
MCHC	33.1 g/dL
RDW%	18.1%
Plt	119 K/CMM
MPV	13.4 fL
*Other*	
D‐dimer	6531 DDu ng/mL

*Note:* Initial laboratory findings on admission. The notable values include an elevated troponin with an unchanged repeat value, an elevated white blood cell count, and elevated D‐dimer.

The patient's home anticoagulation was then switched to intravenous heparin to treat for suspected pulmonary embolism and possible non‐ST‐segment elevation myocardial infarction. The next day, the chest pain resolved, though the patient continued to feel dyspneic. At the time, these symptoms were attributed to the suspected pulmonary embolism and bilateral pleural effusions. To further evaluate the persistent dyspnea and bilateral pleural effusions, a CT scan of the thorax without contrast was performed the following day which showed a dilated aorta at the level of the proximal arch measuring up to 4.5 cm in diameter, a large right pleural effusion, and smaller left pleural effusion. A thoracentesis yielded 500 mL of serosanguinous fluid from the right pleural space with some improvement in dyspnea. Concurrently, the patient's hemoglobin started to trend downward with no clear signs of bleeding such as vascular access site bleeding or hematoma, hemoptysis, hematemesis, or melena. The presence of acute‐onset anemia and continued respiratory distress despite therapeutic anticoagulation and thoracentesis suggested the possibility that another diagnosis was driving the patient's presentation rather than the initial suspicion of pulmonary embolism.

**Figure 1:**
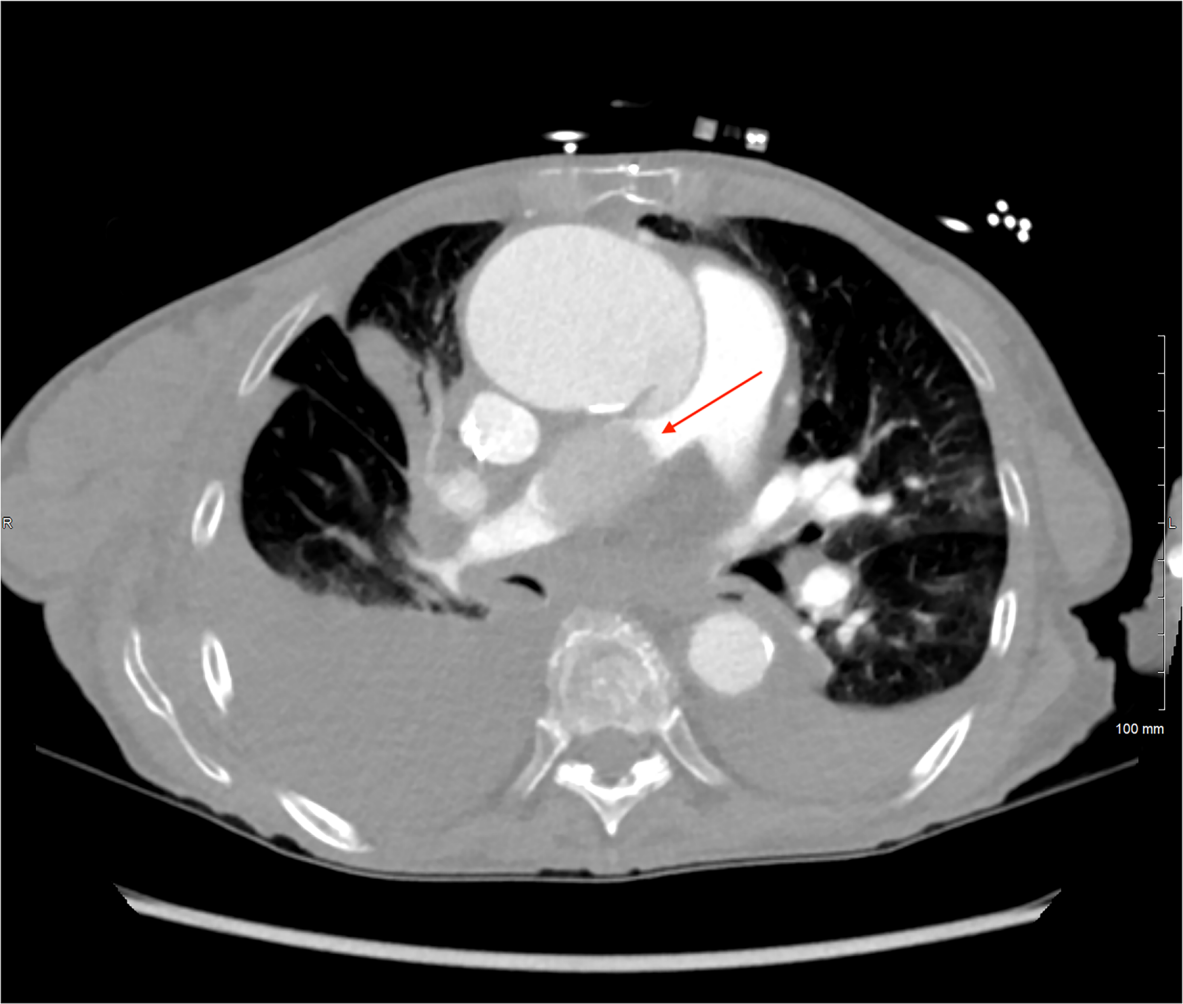
CT angiography of the thorax with pulmonary embolism protocol. This image depicts compression of the right pulmonary artery by the focal mass in the mediastinum measuring 3.1 × 4.3 cm (red arrow) which represents the mediastinal pseudoaneurysm. There is suboptimal filling of the right pulmonary arteries due to the compression. No discrete intraluminal filling defect is seen to suggest pulmonary artery embolism. The cardiac size was enlarged with no pericardial effusion, and reflux of contrast into the hepatic veins was noted, consistent with right heart dysfunction. [Color figure can be viewed at wileyonlinelibrary.com]

**Figure 2:**
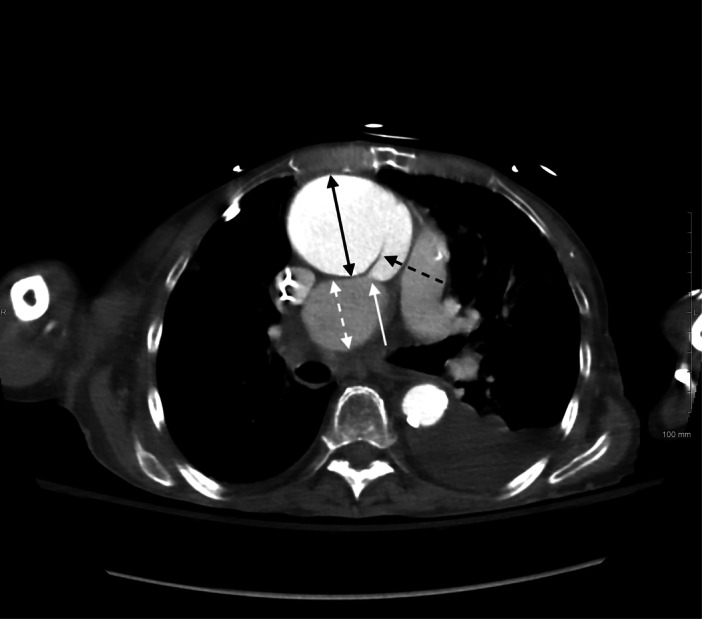
CT angiography of the aorta. There is an aneurysmal dilation of the ascending thoracic aorta measuring up to 6.1 cm in diameter (black double‐sided arrow) with a focal dissection flap (black dashed arrow) in the distal ascending thoracic aorta extending into the arch. There is a focal mass in the mediastinum measuring 3.1 × 4.3 cm (white dashed double‐sided arrow) demonstrating enhancement on postcontrast images that likely represents pseudoaneurysm. Communication between the false lumen of the aortic dissection and focal mediastinal mass is depicted with the white solid arrow.

A CT angiography of the chest with pulmonary embolism protocol, which was performed immediately after noting the acute‐onset anemia, revealed an increase in size of the ascending thoracic aorta with an aneurysmal dilation measuring up to 6.1 cm with an associated focal dissection flap (Figure 1). Also noted was a mediastinal mass measuring up to 4.3 cm in diameter concerning for either a pseudoaneurysm or an intramural hematoma. Notably, there was no discrete intraluminal filling defect to suggest a pulmonary embolism, but the mediastinal mass demonstrated obstruction of the right pulmonary artery. A dedicated aortic CT angiography of the chest was recommended based on these findings and determined that the area of obstruction represented a pseudoaneurysm given that the false lumen of the dissection communicated with the mediastinal mass (Figure 2). The patient was diagnosed with aortic aneurysm with dissection and pseudoaneurysm formation with obstruction of the right pulmonary artery, which explained the acute‐onset anemia and persistent dyspnea and hypoxia despite treatment with anticoagulation. Anticoagulation was promptly discontinued and surgical management versus conservative management were discussed with the patient and family. Unfortunately, the patient's clinical course failed to improve, and respiratory status continued to deteriorate further. The patient and family in conjunction with the treatment team elected to transition to hospice care. The patient passed away shortly after.

## 3 Discussion

This case is one of the only known reports of aortic dissection with pseudoaneurysm formation occurring several weeks after TAVR that presented initially with dyspnea, hypoxia, and intermittent chest pain with initial imaging suggestive of a pulmonary embolism. This case emphasizes the challenges of diagnosing and managing aortic dissection and pseudoaneurysm formation following TAVR, particularly when symptoms are atypical and overlap with more common conditions such as pulmonary embolism. While a direct causal relationship between TAVR and aortic dissection cannot be definitively confirmed in this case, the close temporal association between the procedure, symptom onset, and the patient's moderately controlled blood pressure raises suspicion that the procedure could have contributed to the patient's presentation, though this remains a plausible, rather than confirmed, association.

Aortic dissection following TAVR is an uncommon complication that typically occurs immediately post‐procedure and is detected on peri‐operative imaging [2]. However, pseudoaneurysm formation is an even rarer delayed complication. Several cases in the literature describe late pseudoaneurysm formation after TAVR, some with infectious causes in immunocompromised patients. For instance, a patient with infective endocarditis developed a pseudoaneurysm 1 month after valve‐in‐valve TAVR with a 26‐mm Evolut R valve (Medtronic, NJ, USA). CT imaging identified a pseudoaneurysm, and a positron emission tomography (PET)/CT scan showed abnormal uptake, suggestive of infectious changes [5]. Similarly, another case involved delayed pseudoaneurysm formation over a year after valve‐in‐valve replacement with an Acurate S valve (Boston Scientific, MA, USA) in a patient diagnosed with infective endocarditis. This patient had a significant progression of intraprosthetic regurgitation on the aortic bioprosthesis, with a visible protrusion of the bioprosthesis through the wall of the ascending aorta, causing a pseudoaneurysm [6]. Another notable case involved a lung transplant recipient who developed a pseudoaneurysm 2 years after TAVR with a 34‐mm CoreValve Evolut R valve (Medtronic, NJ, USA). CT imaging showed a retrosternal mediastinal hematoma contiguous with the ascending aorta and a pseudoaneurysm. Friction between the aortic wall and sternal wires, along with the patient's immunocompromised status were thought to have contributed to and increased risk of pseudoaneurysm formation [7]. Lastly, delayed pseudoaneurysm formation was found in a patient 6 months post‐TAVR with a 29‐mm Edwards SAPIEN 3 valve (Edwards Lifesciences, CA, USA). The patient presented with dizziness, a new diastolic murmur, and severe aortic regurgitation, and CT imaging revealed a large pseudoaneurysm [8].

These cases emphasize that pseudoaneurysm formation after TAVR can present with a delayed diagnosis, can be found incidentally, or manifest as symptomatic valve disease. Concomitant infectious process and immunosuppression are linked to cases of aortic pseudoaneurysm post‐TAVR.

Differing from other known reports of aortic dissection and pseudoaneurysm associated with TAVR [2], this case describes a patient who presented 2 weeks after the procedure with symptoms initially suggestive of pulmonary embolism, including dyspnea and intermittent chest pain. The clinical picture was further complicated by presence of bilateral pleural effusions and hypoxia. Coexisting comorbidities and time from procedure made initial diagnostics more difficult, and the initial clinical suspicion of pulmonary embolism more likely. A CT angiography scan, which would have revealed the dissection and resultant aortic pathology that compressed the patient's right pulmonary artery, was initially deferred due to concerns about contrast nephropathy, given the patient's extensive renal history of multiple kidney transplants. However, with relatively preserved renal function (creatinine 0.71 mg/dL with a glomerular filtration rate of >60 mL/minute) and the patient's acute clinical presentation, earlier imaging with a CT angiography could have been justified. The decision to initially defer angiography may have contributed to the delay in diagnosis, and risk of contrast nephropathy was likely overestimated. Additionally, the use of a lung ventilation‐perfusion scan, which was suggestive of a large pulmonary embolism, led to the administration of therapeutic unfractionated heparin. Anticoagulation may have further exacerbated the dissection and pseudoaneurysm, further highlighting the importance of early and accurate imaging in complex cases.

Aortic dissection post‐TAVR is most commonly managed with procedural reintervention (i.e., surgical or endovascular repair) [2]. However, conservative medical management is occasionally considered [2, 3]. The mortality rate of patients sustaining aortic dissection post‐TAVR can be high, reaching up to 66.7% [2]. Furthermore, pleural effusions are a nonspecific finding in some patients with aortic dissection and can prolong time to diagnosis [9].

Several factors increase the risk of aortic dissection following TAVR: female gender, age greater than 80, type A dissection, use of smaller transcatheter valves, and detection during the intraoperative setting. The dissection is commonly caused by posterior annular rupture by the transcatheter valve or transcatheter valve embolization [2]. The patient detailed in our case had many common features among patients who developed aortic dissection post‐TAVR, including older age, female, Stanford type A aortic dissection, and smaller valve used (23‐mm). The patient's immunocompromised status may also have contributed to her predisposition for aortic pathology.

## 4 Conclusion

This case emphasizes the challenges associated with diagnosing and managing rare complications following TAVR, particularly when the presentation is delayed, and symptoms overlap with more common conditions. Despite the relatively stable post‐procedural course, the patient's eventual diagnosis of aortic dissection with pseudoaneurysm formation highlights the importance of maintaining a high index of suspicion for atypical presentations of aortic pathologies even weeks post‐procedure and being aware of barriers in diagnosis that can arise when patients' comorbidities preclude certain imaging modalities. Commonly thought of as an immediate post‐procedural complication, aortic dissection should still be considered despite delays in presentation. Involvement of surrounding structures which may cause atypical symptoms should also be factored into diagnosis. Early recognition through timely imaging and careful consideration of comorbidities is crucial for improving patient outcomes.

## Ethics Statement

Institutional review board (IRB) approval was not required for this single‐patient case report, in accordance with institutional policies and journal guidelines.

## Consent

Written informed consent was obtained before the procedure. All patient identifying information has been removed to protect patient confidentiality.

## Conflicts of Interest

The authors declare no conflicts of interest.

## Data Availability

All relevant clinical data supporting the findings of this case report are included within the article. No additional data are available.

## References

[1] J. D. Carroll , M. J. Mack , S. Vemulapalli , et al., “STS‐ACC TVT Registry of Transcatheter Aortic Valve Replacement,” Journal of the American College of Cardiology 76, no. 21 (2020): 2492–2516, 10.1016/j.jacc.2020.09.595. 33213729

[2] E. Ashwat , D. Ahmad , M. P. Sá , et al., “Acute Aortic Dissection After Transcatheter Aortic Valve Replacement,” American Journal of Cardiology 222 (2024): 108–112, 10.1016/j.amjcard.2024.04.059. 38750948

[3] T. Thomas , A. Poulose , and K. Harris , “Transient Aortic Intramural Hematoma Complicating Transaortic Valve Replacement,” AORTA 04, no. 6 (2016): 232–234, 10.12945/j.aorta.2016.16.029. PMC5425261 28516100

[4] S. T. Chaturvedula , R. Devadoss , and T. Kaneko , “Chronic Aortic Sub‐Adventitial Hematoma Following Supra‐Annular TAVR,” Cardiovascular Revascularization Medicine: Interesting Cases 1 (2024): 100005, 10.1016/j.crmic.2024.100005.

[5] A. Rosenzveig , V. Menon , S. Unai , and G. W. Reed , “Aortic Pseudoaneurysm After Valve‐in‐Valve TAVR,” JACC: Case Reports 29, no. 21 (November 2024): 102692, 10.1016/j.jaccas.2024.102692. 39619043 PMC11602633

[6] V. Vejtasova , J. Bonaventura , R. Topalo , and J. Veselka , “Ascending Aorta Pseudoaneurysm as a Rare, Late Complication After Valve‐in‐Valve Transcatheter Aortic Valve Implantation Procedure,” Acta Cardiologica Sinica 38, no. 5 (September 2022): 642–645, 10.6515/acs.202209_38(5).20220330c. 36176369 PMC9479056

[7] X. Cai , N. Shahandeh , J. Ji , et al., “Ascending Aortic Pseudoaneurysm: A Rare Complication of Transcatheter Aortic Valve Replacement and Thoracic Surgery,” Circulation: Cardiovascular Imaging 15, no. 7 (July 2022): e014076, 10.1161/circimaging.122.014076. 35861982 PMC9304754

[8] L. Salido Tahoces , C. Fernández‐Golfín , A. Pardo Sanz , J. L. Zamorano‐Gómez , and Á. Sánchez Recalde , “Pseudoaneurysm After TAVR: How to Close the Hole?,” European Heart Journal ‐ Cardiovascular Imaging 24, no. 6 (May 2023): e102, 10.1093/ehjci/jead047. 36960604

[9] K. M. Harris , C. E. Strauss , K. A. Eagle , et al., “Correlates of Delayed Recognition and Treatment of Acute Type A Aortic Dissection: The International Registry of Acute Aortic Dissection (IRAD),” Circulation 124, no. 18 (2011): 1911–1918, 10.1161/CIRCULATIONAHA.110.006320. 21969019

[10] J. Bello , Y. Lee , N. Radfar , et al., “PE‐EK A BOO: When PE is Not Really PE ‐ Aortic Dissection with Hematoma Masquerading as Pulmonary Embolism,” supplement, Journal of the American College of Cardiology 83, no. S13 (April 2024): 4126, 10.1016/S0735-1097(24)06116-3.

